# Educate, Nurture, Advise, Before Life Ends Comprehensive Heartcare for Patients and Caregivers (ENABLE CHF-PC): study protocol for a randomized controlled trial

**DOI:** 10.1186/s13063-018-2770-9

**Published:** 2018-08-06

**Authors:** Rachel Wells, Macy L. Stockdill, J. Nicholas Dionne-Odom, Deborah Ejem, Kathryn L. Burgio, Raegan W. Durant, Sally Engler, Andres Azuero, Salpy V. Pamboukian, Jose Tallaj, Keith M. Swetz, Elizabeth Kvale, Rodney O. Tucker, Marie Bakitas

**Affiliations:** 1 School of Nursing, University of Alabama at Birmingham, 1720 2nd Avenue South, Birmingham, AL 35294-1210 USA; 2 Department of Medicine, Division of Gerontology, Geriatrics, Palliative Care, University of Alabama at Birmingham, 1720 2nd Avenue South, Birmingham, AL 35294-1210 USA; 3 Birmingham VA Medical Center, VAMC 11G, 700 19th St South, Birmingham, AL 35233-0001 USA; 4 Department of Medicine, Division of Preventive Medicine, University of Alabama at Birmingham, 1720 2nd Avenue South, Birmingham, AL 35294-1210 USA; 5 Department of Medicine, Division of Cardiovascular Diseases, University of Alabama at Birmingham, 1720 2nd Avenue South, Birmingham, AL 35294-1210 USA; 6 Department of Medicine, Dell Medical School, University of Texas at Austin, 1501 Red River Street, Austin, TX 78712 USA; 7 Department of Medicine, Division of Gerontology, Geriatrics, and Palliative Care, University of Alabama at Birmingham, 1720 2nd Avenue South, Birmingham, AL 35294-2041 USA

**Keywords:** Palliative care, Psychoeducational intervention, Telehealth, Access

## Abstract

**Background:**

Palliative care is specialized medical care for people with serious illness that is focused on providing relief from symptoms and stress and improving the quality of life (QOL) for patients and their families. To help the 6.5 million U.S. adults and families affected by heart failure manage the high symptom burden, complex decision-making, and risk of exacerbation and death, the early integration of palliative care is critical and has been recommended by numerous professional organizations. However, few trials have tested early outpatient community-based models of palliative care for patients diagnosed with advanced heart failure and their caregivers. To address this gap, through a series of formative evaluation trials, we translated an oncology early palliative care telehealth intervention for heart failure to create ENABLE CHF-PC (Educate, Nurture, Advise, Before Life Ends, Comprehensive Heartcare for Patients and Caregivers).

**Methods/Design:**

The primary objective of this multisite pragmatic randomized controlled trial is to test the efficacy of ENABLE CHF-PC plus usual heart failure care compared to usual care alone. Community-dwelling persons who are ≥50 years of age with New York Heart Association class III/IV or American Heart Association/American College of Cardiology stage C/D heart failure and their primary caregiver (if present) are being randomized to one of two study arms. The ENABLE CHF-PC intervention group receives usual heart failure care plus an in-person palliative care assessment by a board-certified palliative care provider (caregivers are invited to attend), a series of nurse coach-led, weekly psychoeducational 20 to 60 min phone sessions using a guidebook called *Charting Your Course* (patients: 6 sessions and caregivers: 4 sessions), and monthly check-in calls. *Charting Your Course* topical content includes problem-solving, coping, self-care and symptom management, communication, decision-making, advance care planning, and life review (patients only). Primary outcomes include patient QOL and mood (depressive symptoms/anxiety) and caregiver QOL, mood, and burden at 8 and 16 weeks after baseline. Outcomes will be examined using an intention-to-treat approach and mixed effects modeling for repeated measures.

**Discussion:**

This trial will determine whether the ENABLE CHF-PC model of concurrent heart failure palliative care is superior to usual heart failure care alone in achieving higher patient and caregiver QOL, improving mood, and lowering burden.

**Trial registration:**

ClinicalTrials.gov, NCT02505425. Registered on 22 July 2015.

**Electronic supplementary material:**

The online version of this article (10.1186/s13063-018-2770-9) contains supplementary material, which is available to authorized users.

## Background

Approximately 6.5 million American adults have received a diagnosis of heart failure (HF) [1]. Despite treatment advances, 22% of patients will die within 1 year of first hospitalization and close to 50% will die within 5 years [1, 2]. By 2030, it is predicted that the prevalence of HF will increase to over 8 million [3]. The morbidity and symptom burden due to HF is often high. Nearly 3 in 4 people suffer from at least one co-morbidity, the most prevalent of which include chronic kidney disease, anemia, diabetes, and chronic obstructive pulmonary disease [4]. The burden of HF is debilitating and extends beyond the patient, who often relies on close family members and friends to assist with medical management and to support their day-to-day activities of living. These tasks can become onerous over time and lead to caregivers experiencing distress and poor mental health [5].

The early integration of palliative care is critical and has been recommended in numerous organizational statements in both the U.S. and worldwide to help persons and families affected by HF manage the high symptom burden, complex decision-making, and ever-present risk of exacerbation and death, [6–15]. The World Health Organization defines palliative care as:

An approach that improves the quality of life of patients and their families facing the problem associated with life-threatening illness, through the prevention and relief of suffering by means of early identification and impeccable assessment and treatment of pain and other problems, physical, psychosocial and spiritual [16].

Palliative care is often mistakenly associated with hospice and end-of-life care [17, 18]. However, the American Heart Association and other professional organizations recommend providing palliative care concurrent with usual HF treatment [10, 15].

Despite these guideline recommendations, there has been little progress in incorporating essential palliative care services into HF care, especially early in the disease trajectory when there is the greatest potential for benefit [18–22]. The 2013 American College of Cardiology Foundation/American Heart Association (ACC/AHA) *Guideline for the Management of Heart Failure* recommended palliative care for all patients with a New York Heart Association (NYHA) class III/IV and ACC/AHA stage C/D. However, only one out of three HF patients receive palliative care [15, 24]. Older patients with HF and their family caregivers (FCGs) rarely have access to palliative supportive care services because the disease trajectory is unpredictable and palliative treatment may not be provided until all other medical treatments have been tried.

While palliative care has been shown to be beneficial in other disease populations, such as cancer [25, 26], there have been few clinical trials to evaluate the early integration of HF palliative care in outpatient and community settings. Observational studies of hospital-based palliative care services report that palliative care consultations may reduce symptom burden and improve quality of life (QOL) [27–30]. Additionally, recent reviews demonstrate decreased healthcare resource utilization with palliative care intervention, although the strongest evidence is for cancer populations [31]. While this is a positive indication of the potential benefits of palliative care, little progress has been made in incorporating these essential palliative care services into HF care. Current elements of recommended HF care management are often lacking, such as self-care, activation, decision support, communication, advance care planning, and care coordination [23, 26, 28, 32–36].

Early concurrent palliative cancer care achieves beneficial outcomes in QOL, symptom burden, depression, and in some cases, survival in oncology patients [37–39]. ENABLE (Educate, Nurture, Advise, Before Life Ends) is a nurse-led multicomponent telehealth care model that provides foundational evidence supporting the World Health Organization continuum of care. It has been mentioned in a recent consensus statement [40] and a Cochrane Systematic Review [41]. The World Health Organization model posited that introducing key palliative care concepts early in the disease trajectory would increase opportunities for patients and families to benefit from all that palliative care has to offer [16]. Unlike hospice care, ENABLE is not prognosis-dependent or focused solely on end of life [42].

In our pilot study, ENABLE CHF-PC (Comprehensive Heartcare For Patients and Caregivers), instruments and procedures from previous ENABLE interventions were successfully adapted for advanced HF patients and their caregivers, thus demonstrating feasibility and acceptability of this model to a predominantly underserved HF population [42]. Therefore, the ENABLE CHF-PC randomized controlled trial (RCT) seeks to determine whether the ENABLE CHF-PC intervention leads to improved self-reported outcomes of QOL, mood, symptom burden, resource use, and caregiver-reported health and burden.

## Methods/design

### Conceptual foundation

The conceptual framework of ENABLE CHF-PC is based on Wagner’s Chronic Care Model (CCM). The CCM is built on collaborative coordinated care, which leads to improved outcomes by promoting self-management and decision support to foster the development of activated empowered patients [43, 44]. Patient activation has been identified as an essential element in improving health-care quality and efficiency, and patient clinical and functional outcomes. Previous interventional studies and meta-analyses using the CCM have demonstrated improved disease-focused HF self-management and survival outcomes [45–53]. Furthermore, based on these previous successes and a study that demonstrated the successful application of the CCM with the ENABLE intervention in advanced cancer [37], ENABLE CHF-PC was successfully pilot tested for advanced HF [42, 54]. ENABLE CHF-PC maintains the original CCM framework by fostering patient activation and empowerment [54] and effective communication about advanced care planning, treatment goals, prognosis, and symptom management [55-57] through a multicomponent coaching approach comprising an in-person palliative care consultation (PCC) [58] and telehealth patient and caregiver sessions. These components are complementary and reinforcing. The in-person PCC provides a guideline-based [58] comprehensive assessment and builds a foundation for future consultation, if needed, and the phone-based coaching sessions provide comprehensive information in an unhurried and convenient home setting. These components are standardized but tailored to individual patient and caregiver needs. The goal of ENABLE CHF-PC is to normalize the early introduction of guideline-recommended [10, 15, 21–23] palliative care content (e.g., dyspnea management, informed decision-making, etc.) by embedding palliative nurse coaches within current HF care. Thus, HF clinicians and patients are exposed to palliative care principles early while concurrently seeking disease-modifying treatment.

### Aims and hypotheses

The study aims are:

1. To determine whether ENABLE CHF-PC leads to a higher level of advanced HF patient-reported QOL and mood (depression/anxiety) [Primary Outcomes] and lower symptom burden and resource use (e.g., hospital admissions and intensive care unit days, and emergency visits)[Secondary Outcomes] at 8 and 16 weeks after baseline.

We hypothesize that intervention participants will experience a higher QOL and mood, and lower symptom burden and resource use (i.e., emergency room visits, intensive care unit days, inpatient days or hospital admissions) at 8 and 16 weeks after baseline compared with those receiving usual HF care.

2. To determine whether ENABLE CHF-PC leads to higher caregiver-reported QOL, mood (anxiety/depression) [Primary Outcomes], and self-reported health [Secondary Outcome] and lower caregiver burden at 8 and 16 weeks after baseline.

We hypothesize that intervention caregivers will report higher QOL, mood, and self-reported health, and lower caregiver burden at 8 and 16 weeks after baseline.

The exploratory aims of the present study are also twofold:

1. To explore mediators and moderators of patient and caregiver outcomes and reciprocal relationships.
2. To examine intervention effects of using joint modeling approaches.

### Setting

The study is a blinded randomized controlled clinical trial conducted at an academic tertiary referral center [University of Alabama at Birmingham (UAB)] and the Birmingham Veterans Affairs Medical Center (BVAMC).

### Study procedures

**Fig. 1:**
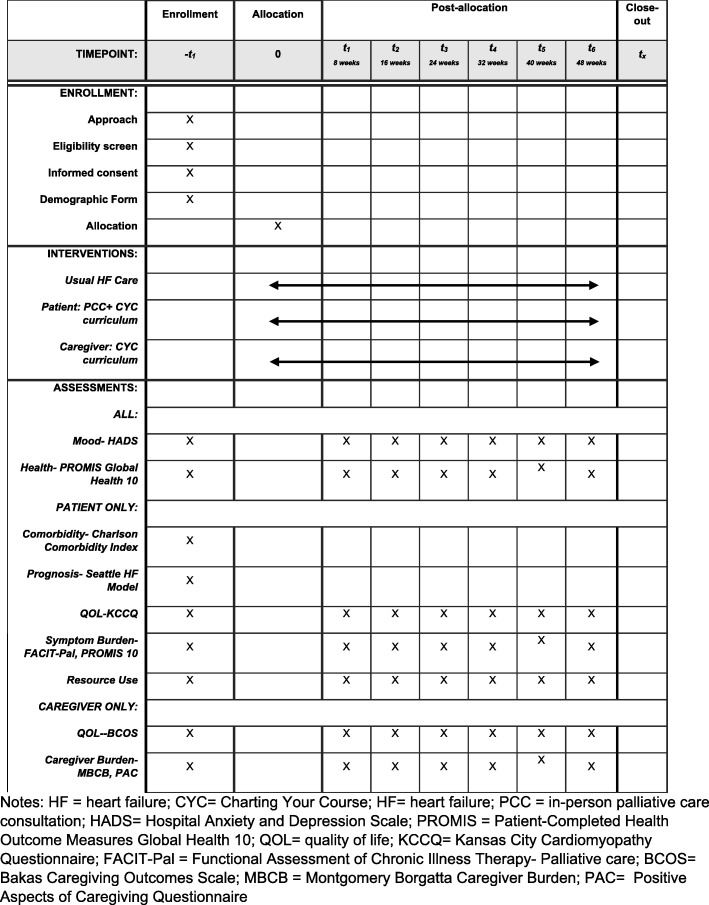
Evaluation of 2013 SPIRIT-recommended content in the ENABLE CHF-PC RCT. CYC Charting Your Course, HF heart failure, PCC in-person palliative care consultation, HADS Hospital Anxiety and Depression Scale, PROMIS Patient-Reported Health Outcome Measures Global Health, QOL quality of life, KCCQ Kansas City Cardiomyopathy Questionnaire, FACIT-Pal Functional Assessment of Chronic Illness Therapy: Palliative Care, BCOS Bakas Caregiving Outcomes Scale, MBCB Montgomery Borgatta Caregiver Burden, PAC Positive Aspects of Caregiving Questionnaire

Study staff target recruitment efforts around potential participants’ scheduled HF clinic visits. After completing a baseline interview, patients are randomly assigned to receive usual HF care or usual HF care plus the ENABLE CHF-PC intervention. FCG participants are assigned to a group based on the patient’s group allocation. The Standard Protocol Items: Recommendation for Intervention Trials (SPIRIT) checklist [59, 60] is included as Additional file 1. The SPIRIT diagram [59] (Fig. 1) depicts time points for enrollment and allocation activities, intervention, and data collection.

### Participants

Specifically, patients meeting the eligibility criteria are recruited from the outpatient UAB HF clinic, the UAB hospital-based transitional care HF clinic, and the BVAMC HF clinic. Patient participants are not excluded if they do not identify a FCG; however, FCGs cannot enroll without concurrent patient enrollment.

Eligibility criteria for patient participants include:

1. Able to speak English
2. Age ≥50 years
3. Clinician-determined NYHA class III/IV or ACC/AHA stage C/D HF [61]
4. Reliable telephone access
5. Able to complete a baseline interview

Exclusion criteria for patient participants includes any of the following:

1. Dementia or significant confusion (measured by Callahan score ≥13) [62]
2. Non-correctable hearing loss
3. Diagnostic and Statistical Manual of Mental Disorders (4th edition) axis I diagnosis (e.g., schizophrenia, bipolar disorder, or active substance use disorder)

The definition of FCG used in the study is broad. FCGs may include a spouse, an adult family member, or a close friend who is considered the primary caregiver and is willing to participate [63].

Eligibility criteria for FCG participants include:

1. Identification by the patient as “a person who knows you well and is involved in your care”
2. Able to speak English
3. Age ≥18 years
4. Able to complete the baseline interview
5. Reliable telephone access

### Recruitment

Recruitment and data collection are conducted by a UAB community-based research and recruitment team. Team members, trained to identify advanced-HF patients, screen UAB and BVAMC cardiology clinic schedules for eligible participants. Potential participants are approached after clinicians’ approval during scheduled outpatient cardiology clinic appointments and eligibility is verified. If the patient agrees to participate, a written consent form is signed. During the informed consent discussion, patients are asked to identify a caregiver for study participation. If the caregivers are present during a clinic appointment, they are consented concurrently with the patients. At UAB, if the caregivers are not present, they are contacted and consented by telephone. At BVAMC, all caregiver approaches and consent must be completed in person. Recruitment began in October 2015.

### Randomization and blinding

After consent is obtained, a baseline interview is completed by phone. Patients are stratified by center (UAB or BVAMC), referring service (HF clinic, cardiology, or other ), and race (Caucasian or non-Caucasian). Patients are randomized using a 1:1 ratio to either intervention or usual care. The study program manager is responsible for randomization and verifies a computer-generated treatment assignment with a randomization table. A study staff member notifies participants about their allocation status. Data collectors are blinded regarding participants’ group assignment. Due to the nature of the study, participants and nurse coaches are aware of allocation to the intervention group.

### Data collection

A secure REDCap (Research Electronic Data Capture) database is used to capture all data collection points. The data collection section of the database consists of three parts: (1) screened participants’ contact information, (2) call attempt log, and (3) collected data measures. Trained research personnel collect patient- and caregiver-reported data by phone which is then directly entered into the REDCap database. Patient- and caregiver-reported outcome measures are collected every 8 weeks for 48 weeks. Primary outcome measures collected for patient participants include QOL and mood. Primary outcome measures collected for caregiver participants include QOL, mood, and burden. Secondary patient outcome measures collected include health status, symptom burden, and resource use. Secondary caregiver outcome measures include self-reported health. Exploratory outcomes include mediators, moderators, and reciprocal relationships [e.g., Patient Assessment of Chronic Illness Care (PACIC), dyadic HF typology, and dyadic adjustment]. Additionally, health literacy, demographic information, spiritual and religious coping, and reciprocal relationships are measured for both participants and caregivers at baseline. Patient and caregiver outcome measures are described in detail in Table 1.

**Table 1:** ENABLE CHF-PC randomized controlled trial outcome measures

	Construct	Instrument	Description of measure	Reliability	Schedule
Specific aim 1					
HF patients	QOL	Kansas City Cardiomyopathy Questionnaire (KCCQ)	5 domains: physical limitations, symptoms, self-efficacy, social interference, and QOL; 23 items	Subscales α =0.62 to 0.90	Baseline + every 8 weeks for 48 weeks
	Mood	Hospital Anxiety and Depression Scale (HADS)	2 domains measuring depression and anxiety; 14 items	Subscales α = 0.82 to 0.83	
	Symptom burden	FACIT-Pal (14-item)	4 domains: physical, social/family, emotional, and functional well-being; 14 items	Subscales α = 0.75 to 0.93	
		PROMIS pain intensity scale; 3-item SF and pain interference (2 items)	3-item PROMIS pain intensity scale; 3 items; patient responses are from 1 (had no pain) to 5 (very severe); PROMIS pain interference; 2 items	α = 0.33 to 0.93 (intensity); NA (interfere)	
	Resource use	Patient resource use	Inpatient days, ICU days, ED visits, hospice use, palliative care provider visits, AD completion, DNR orders	NA	
Specific aim 2					
Caregivers	QOL	Bakas Caregiving Outcomes Scale (BCOS)	15-item scale that measures changes in social functioning, subjective well-being, and somatic health of caregiver	Subscales α = 0.72 to 0.90	Baseline + every 8 weeks for 48 weeks
	Mood	HADS	(same as above)	(same as above)	
	Caregiver burden	Montgomery Borgatta Caregiver Burden Scale	Measure of caregiver burden with 3 domains: objective burden, stress burden, and demand burden; 14 items	Subscales α = 0.75 to 0.88	
		Positive Aspects of Caregiving (PAC)	Measure of caregiver’s mental–affective state in relation to the caregiving experience; 9 items	Subscales α = 0.85 to 0.88	
Exploratory aims					
HF patients	Activation	Patient Assessment of Chronic Illness Care (PACIC)	5 dimensions: activation, delivery system/decision support, goal-setting, problem-solving, and coordination; 20 items	Subscales α = 0.62 to 0.90	Baseline + every 8 weeks for 48 weeks
	Coping style	Brief cope	2 subscales: active and avoidant coping; 28 items	α = 0.68 to 0.79	Baseline only
	Social support	Multidimensional Scale of Perceived Social Support (MSPSS)	Perceived adequacy of support from family and friends; 12 items	α = 0.81	Baseline only
Both	Health literacy	Rapid Estimate of Adult Literacy in Medicine	Measure of health literacy and numeracy based on ability to recognize common health-related terms; 7 items	α > 0.80	Baseline only
	Self-reported health	PROMIS SF Global Health 10	2 domains: physical and mental health; 10 items	Subscales α = 0.81 to 0.86	Baseline + every 8 weeks for 48 weeks
	Demographics	Demographic questionnaire	Age, gender, race, marital status, religion, education, occupation, health insurance, smoking, etc.	NA	Baseline only
	Spiritual and religious coping	Brief multidimensional measure of religiousness and spirituality	Assesses two patterns of religious and spiritual coping with stressful life events: positive religious and spiritual coping reflective of benevolent religious methods of understanding and dealing with life stressors; and negative religious and spiritual coping reflective of religious struggle in coping; 7 items	Subscale α = 0.83	Baseline + every 8 weeks for 48 weeks
	Reciprocal relationships	Dyadic HF care typology	Four basic categories of typology for dyad	NA	Baseline only
		Dyadic adjustment scale- SF	Measures degree of agreement on relational factors such as shared philosophy, goals and time spent together; 7 items	Subscales for patients: α = 0.70; for caregivers: α = 0.78	Baseline + every 8 weeks for 48 weeks

*QOL* quality of life, *BCOS *Bakas Caregiving Outcomes Scale, *NA* not applicable, *FACIT-Pal* Functional Assessment of Chronic Illness Therapy: Palliative Care, *HADS* Hospital Anxiety and Depression Scale, *HF* heart failure, *ICU* intensive care unit, *KCCQ* Kansas City Cardiomyopathy Questionnaire, *ED* emergency department, *AD* advance directive, *DNR* do not resuscitate, *MSPSS* Multidimensional Scale of Perceived Social Support, *PAC* Positive Aspects of Caregiving, *PACIC* Patient Assessment of Chronic Illness Care, *PROMIS* Patient-Reported Health Outcome Measures Global Health, *SF* Short Form

### Intervention

**Fig. 2:**
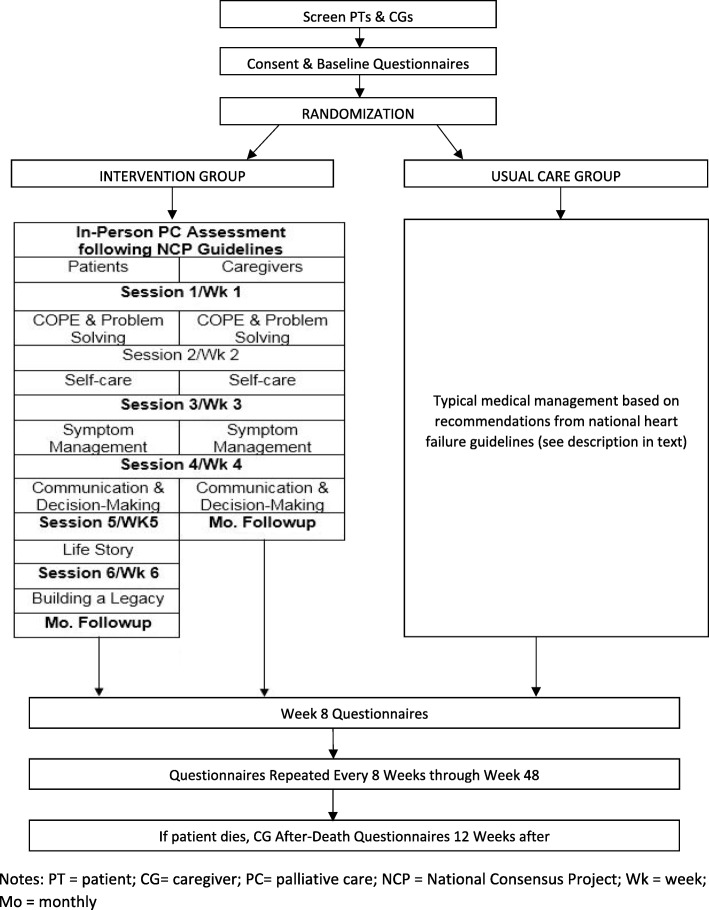
ENABLE CHF-PC study flow chart and CYC content. CYC *Charting Your Course*, PT patient, CG caregiver, PC palliative care, NCP National Consensus Project, Wk week, Mo monthly

The ENABLE CHF-PC intervention includes two major components: (1) an in-person comprehensive PCC as soon as feasible after enrollment (performed at the participants’ study site, e.g., UAB or BVAMC supportive care clinic) and (2) telephone-based nurse coaching sessions following a six-session patient curriculum and a four-session caregiver curriculum followed by monthly supportive care check-in phone calls through the end of data collection (48 weeks) or patient death. The nurse coach interacts with the patient and FCG over time and across settings (home, clinic, hospital, and hospice) via telephone using the standardized curriculum and guidebook: *Charting Your Course (CYC): An Intervention for Patients with Heart Failure and their Families* (Fig. 2). The CYC materials are mailed to participants prior to the first session. Participants are asked to review each chapter prior to the session, but this is not required. Based on our prior work and recent pilot study [42, 54], sessions last approximately 30 to 40 min. After notification of intervention allocation, the nurse coaches complete an introductory phone call to the patient or caregiver and confirm receipt of the CYC materials and schedule the first weekly session. The nurse coaches conduct the scheduled weekly sessions, directly entering field notes into the REDCap database, and then continue to follow participants monthly to check on patient and caregiver needs and to reinforce prior content.

PCC and nurse coach involvement is complementary; the in-person PCC provides expert symptom assessment and the nurse coaches’ sessions deliver comprehensive palliative care and HF information in a convenient setting. The primary goal of the intervention is to encourage patient empowerment; however, occasionally the nurse coaches may provide feedback directly to the HF teams (or palliative care teams) about specific issues (e.g., unrelieved pain) or makes referrals to other resources. The HF teams and palliative care teams are responsible for direct medical care. If an intervention patient is hospitalized, they will be followed by the attending service and a PCT consultation will be offered. Nurse coaches also work with participants to identify their local community resources or assist them in locating them, continuing to reinforce the community-based focus of the intervention.

### Usual care

Patient participants randomized to the usual care group receive their cardiology-related outpatient care consistent with their care prior to enrolling in study. Typical HF patient medical management is based on national HF guidelines [10]. At UAB, HF patients randomized to the usual care group receive their outpatient care through the Advanced HF or Cardiology Clinics at the UAB Kirklin Clinic or the Cardiology Clinic located within UAB Hospital. At the BVAMC, HF patients are supervised by primary care, general cardiology, and geriatrics clinicians. Primary care practices are located in patients’ local communities. Hospitalized patients are managed by the admitting service (cardiology or hospitalist). PCC are rare, but may be offered at the discretion of the clinician.

### Training and treatment fidelity monitoring

To ensure fidelity of the intervention, we employ multiple strategies to address standardized provider training, measurement of treatment delivery, and evaluation of participant receipt. While experience of palliative care, cardiac, and psychiatric nursing was welcomed, it was not required for the nurse coach position. The nurse coaches are trained by the study staff, which includes 28 h of didactic and interactive role-play guided by an established training manual including specific intervention skills (e.g., problem-solving, decision support, and health coaching). Intervention experts review the digitally-recorded mock training sessions and provide feedback until the nurse coaches are confident in their skills. During intervention delivery, the nurse coaches enter field notes into the REDCap database in a template that corresponds to both the standardized script and to a previously developed fidelity checklist adapted to this study. Nurse coaches also participate in weekly meetings with experts to discuss ongoing intervention delivery experience and issues encountered during weekly participant calls. These weekly meetings also act as booster sessions. Expert external reviewers review 10% of all digitally recorded coaching sessions, stratified by participant type, site, and nurse coach. The sessions are scored using fidelity checklists and nurse coaches are provided a copy of each scored session with both core component scores and an overall session score. Nurse coaches who exhibit a pattern of non-adherence (<80%) on three consecutive ratings receive additional training and supervision. External reviewers review all PCC notes, which are recorded in the electronic medical record using a fidelity template comprising PCC elements based on National Consensus Project guidelines [58]. Clinicians completing the consultations are given aggregate feedback based on the template. Following the collection of outcome measures at week 48, all intervention participants will be interviewed by phone to determine the extent of treatment receipt. These interviews are recorded digitally.

### Statistical analysis plan

#### Specific aims

Primary data analysis will begin by examining the balance between groups with descriptive statistics and measures of effect size for baseline characteristics and outcomes by treatment group. For patients, we will conduct longitudinal (intention-to-treat) analyses of the primary study outcomes (QOL and mood) for participants with baseline and one or more follow-up assessments using mixed effects modeling for repeated measures to examine the relative impact of ENABLE CHF-PC at 8 and 16 weeks after baseline. To be considered positive for patients and caregivers, statistically significant effects will need to be seen in patient QOL and mood and caregiver QOL, mood, and burden, respectively. Linear contrasts will be used to estimate the average post-intervention change for each group and for conducting tests of between-group differences. Standardized intervention effect sizes will be estimated for each outcome using baseline standard deviations. A false discovery rate criterion will be used to correct for multiple significance testing. Caregiver outcomes will be analyzed using the same longitudinal methods as outlined above. Only decedents’ caregivers will have grief outcomes compared between groups, which will not involve longitudinal modeling.

#### Exploratory aims

Previously cited palliative care studies have shown alterations in both QOL and survival even though the interventions are not directly intended to impact survival. Therefore, we will compare differences in survival between groups using Kaplan-Meier plots and log-rank tests. Using path analytic models, we will examine whether the collected data support a hypothesized mediation mechanism in which the intervention effect on patient QOL, mood, and symptom burden is mediated by the quality of chronic illness care (PACIC), active and avoidant coping styles (COPE), social support (Multidimensional Scale of Perceived Social Support), and health literacy (Newest Vital Sign). All analyses will be performed using the latest versions of SAS and R.

#### Sample size calculation

We estimated the sample size for the specific aims assuming a time-averaged [64] between-group standardized minimum detectable difference of *d* = .33 (with five time-points, every 8 weeks from week 16 through 48 post-enrollment). Assuming an intra-subject correlation of 0.5 and a significance level of 0.01, a sample size of 130 per group is necessary to achieve 80% power. After accounting for loss to follow-up of 30% due to either death (20%) or independent attrition (10%), the target patient sample size was estimated at 190 per group (total *N*_patient_ = 380) and 114 per caregiver group (total *N*_caregiver_ = 228) with an overall participant total of 608.

#### Ethical aspects

This study has been approved by the institutional review boards at UAB and BVAMC and registered on clinicaltrials.gov (NCT02505425). An independent data safety monitoring committee composed of UAB School of Nursing staff monitor the collection and analysis of data annually, including reviews of blind-protected preliminary analyses. All databases are secure, HIPPA-compliant, and password protected for both user front-end access and back-end storage. The back-end data is housed on a secure drive with access limited to only authorized research personnel. Study modifications are discussed with all investigators and research coordinators until consensus is reached. Amendments reflecting study modifications are submitted for institutional review board approval at all sites and updated on the clinical trial registry. The initial study protocol (version 1.0) was approved in 2014 and underwent minor modifications (version 1.1), which were approved in September 2015.

## Discussion

Despite strong evidence for palliative treatments such as opioids for pain and dyspnea, psychotherapy, antidepressants, and multicomponent counseling interventions [30], few are part of routine HF care [10, 25]. There is growing evidence that providing palliative care early and concurrent with routine HF care can improve HF symptoms and QOL [10, 26, 38, 72], relieve physical and emotional suffering [19, 65–69], and possibly reduce hospitalizations [70–72]. However, there are currently few care models that consistently provide this care and little is known about age-dependent HF outcomes or treatment response because clinical exclusion criteria favor younger patients with less co-morbidity.

ENABLE CHF-PC is an innovative RCT that addresses the urgent need to integrate palliative care into the routine care of underserved older adults with HF in the following ways. First, ENABLE CHF-PC will be the first pragmatic RCT to study an extensively tested evidence-based telehealth palliative care coaching intervention tailored to older underserved adults. It introduces novel problem-solving, decision support techniques and patient decision aids, and life review early in the advanced-HF trajectory as recommended by the 2013 ACC/AHA Guideline [15]. Second, ENABLE CHF-PC has a specific caregiver component, with a special focus on caregiver health literacy and self-reported health. Third, this RCT will contribute to palliative care science by examining factors that may impact patient and caregiver intervention mechanisms (e.g., health literacy, coping, and social support) and novel analytic methods that can inform future intervention development and testing. Finally, this multi-site study brings together an established inter-professional investigative team representing palliative care, HF, geriatrics, sociology, and behavioral psychology within a research-intensive infrastructure to address the special needs of underserved older adults with HF and their family caregivers.

Given the innovative nature of this early palliative care RCT for advanced-HF patients and caregivers, we have planned a multi-pronged approach to dissemination. Findings from this study will be disseminated through multiple peer-reviewed publications and presentations at national and international palliative care and cardiology conferences. Additionally, we plan to share our results through institutional websites and social media.

### Trial status

ENABLE CHF-PC is an ongoing early palliative care RCT. Recruitment began in October 2015. At the time of submission of this manuscript, recruitment and randomization are actively ongoing and will continue through July 2018.

## Additional file

Additional file 1: SPIRIT 2013 Checklist: Recommended items to address in a clinical trial protocol and related documents. (DOC 123 kb)

## Abbreviations

ACC: American College of Cardiology
AD: Advance directive
AHA: American Heart Association
BCOS: Bakas Caregiving Outcomes Scale
BVAMC: Birmingham Veterans Affairs Medical Center
CCM: Chronic Care Model
CG: Caregiver
CYC: Charting Your Course
DNR: Do not resuscitate
ED: Emergency department
ENABLE: Educate, Nurture, Advise, Before Life Ends
ENABLE CHF-PC: Educate, Nurture, Advise, Before Life Ends: Comprehensive Heartcare for Patients and Caregivers
FACIT-Pal: Functional Assessment of Chronic Illness Therapy: Palliative Care
FCG: Family caregiver
HADS: Hospital Anxiety and Depression Scale
HF: Heart failure
ICU: Intensive care unit
KCCQ: Kansas City Cardiomyopathy Questionnaire
Mo: Monthly
MSPSS: Multidimensional Scale of Perceived Social Support
NA: Not applicable
NCP: National Consensus Project
NIH: National Institutes of Health
NINR: National Institute of Nursing Research
NYHA: New York Heart Association
PAC: Positive Aspects of Caregiving
PACIC: Patient Assessment of Chronic Illness Care
PC: Palliative care
PCC: Palliative care consultation
PROMIS: Patient-Reported Health Outcome Measures Global Health
PT: Patient
QOL: Quality of life
RCT: Randomized controlled trial
REDCap: Research Electronic Data Capture
SPIRIT: Standard Protocol Items: Recommendation for Intervention Trials
UAB: University of Alabama at Birmingham
